# Artificial Intelligence-Assisted ECG in a Hub-and-Spoke Network in India: Real-World Performance in Acute Coronary Syndrome Detection and Diagnostic Turnaround Times

**DOI:** 10.7759/cureus.104518

**Published:** 2026-03-01

**Authors:** Praveen Chandra, Aditya Batra, Anupam K Singh, Prakash Chandwani, Pranav Shamraj, Suvir Gupta, Vijaysinh Patil, Shital Sarda

**Affiliations:** 1 Cardiology, Medanta - The Medicity, Gurgaon, IND; 2 Cardiology, Holy Heart Hospital, Rohtak, IND; 3 Cardiology, Sparsh Clinic, Ranchi, IND; 4 Cardiology, CKS Hospitals, Jaipur, IND; 5 Cardiology, Bhaktshreshtha Kamalakarpant Laxman (BKL) Walawalkar Hospital, Ratnagiri, IND; 6 Cardiology, Global Heart Institute, Agra, IND; 7 Cardiology, Krishna Institute of Medical Sciences, Karad, IND; 8 Cardiovascular, Renal, and Metabolism (CVRM), AstraZeneca Pharma India Limited, Bengaluru, IND

**Keywords:** acute coronary syndrome, artificial intelligence, electrocardiogram (ecg/ekg), hub-and-spoke model, india, patient care

## Abstract

Introduction: Acute coronary syndrome (ACS) remains a leading cause of morbidity and mortality in India, with delayed diagnosis contributing to poor outcomes. The ‘Heart Beat’ project - an AI-assisted hub-and-spoke model was implemented to improve early and accurate ACS detection across diverse healthcare settings. In this retrospective, real-world observational analysis, we leveraged data from this initiative to (1) assess the prevalence of cardiovascular diseases (CVD) in the Indian population using AI-powered electrocardiogram (ECG) interpretation and (2) evaluate the turnaround time (TAT) for ECG performance and diagnosis.

Methods: This report utilized data from the Heart Beat project, collected between January 2020 and December 2022. We included patients presenting with chest pain at primary healthcare centers (spokes), linked to centrally located hospitals (*hubs*) equipped with a catheterization laboratory (Cath-lab). AI-assisted 12-lead ECGs were performed at spokes, and data were transmitted to hubs for centralized analysis. ECG findings were categorized as Abnormal, Borderline, Critical, Normal, Otherwise Normal, or Pacemaker based on AI interpretation. Descriptive statistics were employed to evaluate (1) the prevalence of CVD patterns and (2) the TAT for ECG acquisition-to-diagnosis. Findings were analyzed using descriptive statistics.

Results:A total of 45,488 ECGs were obtained from 66 spokes, which were connected to 10 hubs across six Indian states. The most commonly reported ECGs were Normal (n=22987, 50.53%), Abnormal (n=19396, 42.64%), and Critical (n=2992, 6.58%). Most of the CVD conditions were diagnosed from Critical and Abnormal ECGs. Out of the seven CVD conditions analyzed from the dataset, left ventricular hypertrophy (LVH; n=3993, 8.78%) was the most frequently reported. Higher ST-elevation myocardial infarction (STEMI) was identified in 231 cases (0.51% of total ECGs), whereas non-STEMI (NSTEMI) was identified in 22 cases (0.05% of total ECGs) among the total ECGs recorded. The mean (SD) TAT for ECGs of all patients was 5.12 (9.61) minutes, with the minimum mean TAT recorded for Critical (2.91 minutes) and the maximum for Normal (5.61 minutes) ECGs.

Conclusion: The findings highlight a higher prevalence of STEMI over NSTEMI cases, emphasizing the need for adequate resources at healthcare centers for timely and effective management. An average lower ECG TAT (5 minutes) further supported AI-assisted ECG's potential in the early diagnosis of ACS patients.

## Introduction

Acute coronary syndrome (ACS) includes conditions such as ST-segment elevation myocardial infarction (STEMI), non-ST-segment elevation myocardial infarction (NSTEMI), and unstable angina, all of which arise from a sudden decrease in blood flow to the heart, resulting in a heart attack [1]. Cardiovascular diseases (CVD), including ACS, are among the leading causes of death globally [2], responsible for approximately 19 million deaths each year, with ischemic heart disease (IHD) contributing to ~9 million of these fatalities [3,4]. Notably, Asia accounts for about 60% of the world’s population and bears 50% of the global CVD burden, with ACS a major contributor to the region’s mortality [5,6]. In India, where CVD accounts for 71.13 million cases and 27% of deaths according to the 2019 Global Burden of Diseases study, the death rate from CVD surpasses the global average (282 vs. 233 per 100,000) [7,8]. Furthermore, Indian patients develop coronary artery disease at younger ages (~59.6 years) compared to global averages (64.3 years) [9], and experience their first episode of heart attack earlier than European patients (53 vs. 63 years) [10].

About 30% of ACS patients globally are misdiagnosed or left untreated, which significantly increases mortality [11,12]. Furthermore, only 59% of heart attack patients are referred for cardiac rehabilitation, an essential component of post-ACS care [13]. For STEMI patients, guidelines recommend a door-in-door-out (DIDO) time of ≤30 minutes to ensure prompt treatment [14,15], though only about 1.3% of cases worldwide meet this target [16]. Mortality rates are also reduced when STEMI patients reach the hospital within 90 minutes of symptom onset [17]. Yet, in India, the typical arrival time is considerably longer, between 180 and 330 minutes, compared to 110-140 minutes in the USA [18]. This delay is often attributed to a shortage of specialists, limited availability of electrocardiogram (ECG) services or delays in the interpretation of ECGs at primary health centers, lack of symptom awareness, longer travel distances, and poor transportation infrastructure [18-21].

Approximately 70% of India’s population lives in rural areas, while 75% of qualified consultants practice in urban centers, leading to significant healthcare disparities [22,23]. To address this, the Government of India has introduced the hub-and-spoke model, a system used to improve healthcare access in several countries [22,24-26], and positive outcomes are already evident in some Indian states [24]. In this model, when a patient with chest pain reaches a primary care center (spoke), an ECG is performed and sent via cloud networking to a tertiary center (hub), where an expert cardiologist interprets the results. Based on the diagnosis, patients are quickly directed to the nearest Cath Labs or fibrinolysis centers for timely treatment.

Project Heart Beat is a large-scale implementation of the hub-and-spoke model clubbed with artificial intelligence (AI)-assisted ECG diagnosis, initiated in the Indian tier I and II towns and villages that lack access to quality healthcare. The main objective of this program is to aid in an early and accurate diagnosis of ACS in patients presenting with chest pain in a primary care facility. This report presents the initial analysis of the project data, with mainly two objectives: to assess the prevalence of CVD, including STEMI, arrhythmias, and structural abnormalities, across the Indian population using AI-powered ECG interpretation within a hub-and-spoke healthcare model; and to evaluate the turnaround time (TAT) for ECG performance and diagnosis, with emphasis on critical cases requiring urgent intervention (e.g., STEMI, where delays directly impact mortality).

## Materials and methods

The hub-and-spoke model of the Heart Beat project consisted of hospitals with a Cath Lab designated as 'hub' and clinics, nursing homes, diagnostic centers, or those hospitals without a Cath Lab designated as 'spoke.' While more hubs were present in the Heart Beat project dataset, the current analysis is based on data from 10 hubs and 66 spokes across six Indian states. The hub centers are well equipped with adequate infrastructure and skilled technologists/healthcare professionals (HCPs) to treat ACS cases. Data from any patient seeking medical attention at the spokes for chest pain were considered eligible for inclusion in the analysis.

Data collection and cloud-based transmission to hub centers

Using a cloud-connected 12-lead ECG device (Tricog), a skilled technician performed initial examinations of patients with chest pain upon arrival at the spoke center (between January 2020 and December 2022). The collected ECG data were securely sent to the Tricog cloud-based centralized hub via mobile or wifi network. There, Tricog’s AI-based algorithm generated an ECG report, which was sent to doctors at the hubs. Subsequently, a detailed second diagnosis by experts at the hub resulted in the generation of the final ECG analysis report, which was transmitted back to the spokes via mobile app, email, or the customer portal. The critical cases were notified to the hub through an app. The Tricog’s device works on the basic algorithm Algo2 version 0.28.0.

Overall architecture of Tricog’s AI engine

All ECG recordings were processed through automated noise-reduction and signal-processing steps. The AI engine performed both beat-based and lead-based (based on the average or median beat) analyses to evaluate dominant ECG patterns and detect morphological variations, including sporadic events such as premature ventricular complexes (PVCs). Multiple classifier models, including rule-based systems, machine-learning models, neural-network-based classifiers, and deep learning architectures, were trained using ECG features and in-house physicians. The AI system generated preliminary diagnostic outputs along with key ECG features, which were subsequently reviewed by trained physicians. Final physician-validated ECG reports were transmitted to the remote site within minutes of ECG capture.

Endpoints and statistical analysis

Data on different categories of ECGs, such as Abnormal, Borderline, Critical, Normal, Otherwise Normal, and Pacemaker ECGs, were collected through the Heart Beat project. These categories were defined as follows: Abnormal: an ECG showing sinus rhythm with tachycardia (SRTH) and left ventricular hypertrophy (LVH); Borderline: an ECG showing SRTH and left atrial enlargement or SRTH and right atrial enlargement; Critical: an ECG showing life-threatening rhythm disturbances or acute ischemic changes that require immediate medical attention. Examples include findings such as acute myocardial infarction and ventricular tachycardia; Normal: an ECG showing normal sinus rhythm; Otherwise Normal: an ECG with sinus bradycardia or sinus tachycardia with premature ventricular contractions. The incidence of potential cardiac abnormalities, including STEMI, NSTEMI, left bundle branch block (LBBB), atrial fibrillation (AFIB), left ventricular hypertrophy (LVH), suspected ACS, and ventricular tachycardia (VT), was assessed from the collected ECGs. The proportions of different CVD conditions across ECG categories were analyzed both for the overall dataset and for individual hub-wise datasets. Data were analyzed descriptively and presented as n (%). This was a retrospective analysis of routinely collected program data; therefore, no a priori sample size calculation was performed. The analytical sample represents a convenience sample comprising all eligible ECGs captured within the Heart Beat network during the prespecified study window (January 2020 to December 2022). Finally, TAT, defined as the time from the patient’s entry into the spoke center to ECG report generation, was analyzed for overall data and each ECG category. All analyses were done using SAS software (SAS 9.4; SAS Institute Inc., Cary, North Carolina).

Ethical considerations

Patient consents were recorded during the report generation at the healthcare centers. No patient identification data were used for the purpose of this publication. No personal data was shared with any third parties. Institutional ethics committee oversight was waived, as no patient interventions occurred.

## Results

In this analysis, the hub-and-spoke network connected a single hub to a maximum of nine and a minimum of three spokes (Table 1). As shown in Table 2, a total of 45,488 ECGs were performed, with 50.5% (22,987/45,488) considered Normal, 42.6% (19,396/45,488) Abnormal, and 6.58% (2992/45,488) Critical ECGs. The maximum of these ECGs was recorded in the state of Maharashtra (Table 3). All ECG classifications (Normal/Abnormal/Critical) reported in this analysis represent AI-generated diagnoses by Tricog’s central hub platform, which were used for clinical triage in the studied network.

**Table 1:** Details for hubs, clinics, and the recorded ECGs across different states ECG, electrocardiogram.

Hub No. (IDs)	Clinics	States	Total ECGs recorded, N=45488; n (%)
5338	9	Maharashtra	15632 (34.37)
10497	9	Maharashtra	
16147	8	Haryana	13251 (29.13)
10529	6	Haryana	
16180	4	Haryana	
21566	9	Haryana	
10197	9	Madhya Pradesh	8329 (18.31)
14805	3	Rajasthan	2672 (5.87)
21578	6	Uttar Pradesh	4838 (10.64)
16552	5	Jharkhand	766 (1.68)

**Table 2:** Recorded ECGs and ECG turnaround time (TAT) ECG, electrocardiogram; SD, standard deviation; TAT, turnaround time.

ECG categories	Total ECGs recorded (N=45488) n (%)	TAT for ECG mean (SD)
Overall	45,488	5.12 (9.61)
Abnormal	19,396 (42.64)	4.88 (8.81)
Borderline	10 (0.02)	5.00 (4.37)
Critical	2992 (6.58)	2.91 (4.85)
Normal	22,987 (50.53)	5.61 (10.65)
Otherwise Normal	38 (0.08)	3.50 (3.40)
Pacemaker	65 (0.14)	2.95 (3.04)

**Table 3:** Summary of ECG data by states Note: Percentages are relative to the total dataset (N=45,488). ECG, electrocardiogram.

Hub No.	State	Total ECGs recorded n (%)	Abnormal n (%)	Borderline n (%)	Critical n (%)	Normal n (%)	Otherwise Normal n (%)	Pacemaker n (%)
5338	Maharashtra	10,362 (22.80)	4807 (10.57)	02 (0.00)	473 (1.04)	5,065 (11.13)	08 (0.02)	07 (0.02)
10497	Maharashtra	5,270 (11.60)	2317 (5.09)	00	703 (1.55)	2244 (4.93)	00	06 (0.01)
16147	Haryana	4,319 (9.49)	1847 (4.06)	00	163 (0.36)	2,308 (5.07)	00	01 (0.00)
10529	Haryana	6,719 (14.80)	2563 (5.63)	08 (0.02)	329 (0.72)	3,788 (8.33)	30 (0.07)	01 (0.00)
16180	Haryana	90 (0.20)	59 (0.13)	00	06 (0.01)	24 (0.05)	00	01 (0.00)
21566	Haryana	2,123 (4.67)	866 (1.90)	00	155 (0.34)	1,100 (2.42)	00	02 (0.00)
10197	Madhya Pradesh	8,329 (18.30)	3411 (7.50)	00	495 (1.09)	4423 (9.72)	00	00
14805	Rajasthan	2,672 (5.87)	1099 (2.42)	00	144 (0.32)	1,423 (3.13)	00	06 (0.01)
21578	Uttar Pradesh	4,838 (10.60)	2101 (4.62)	00	467 (1.03)	2,242 (4.93)	00	28 (0.06)
16552	Jharkhand	766 (1.68)	326 (0.72)	00	57 (0.13)	370 (0.81)	00	13 (0.03)

**Figure 1:**
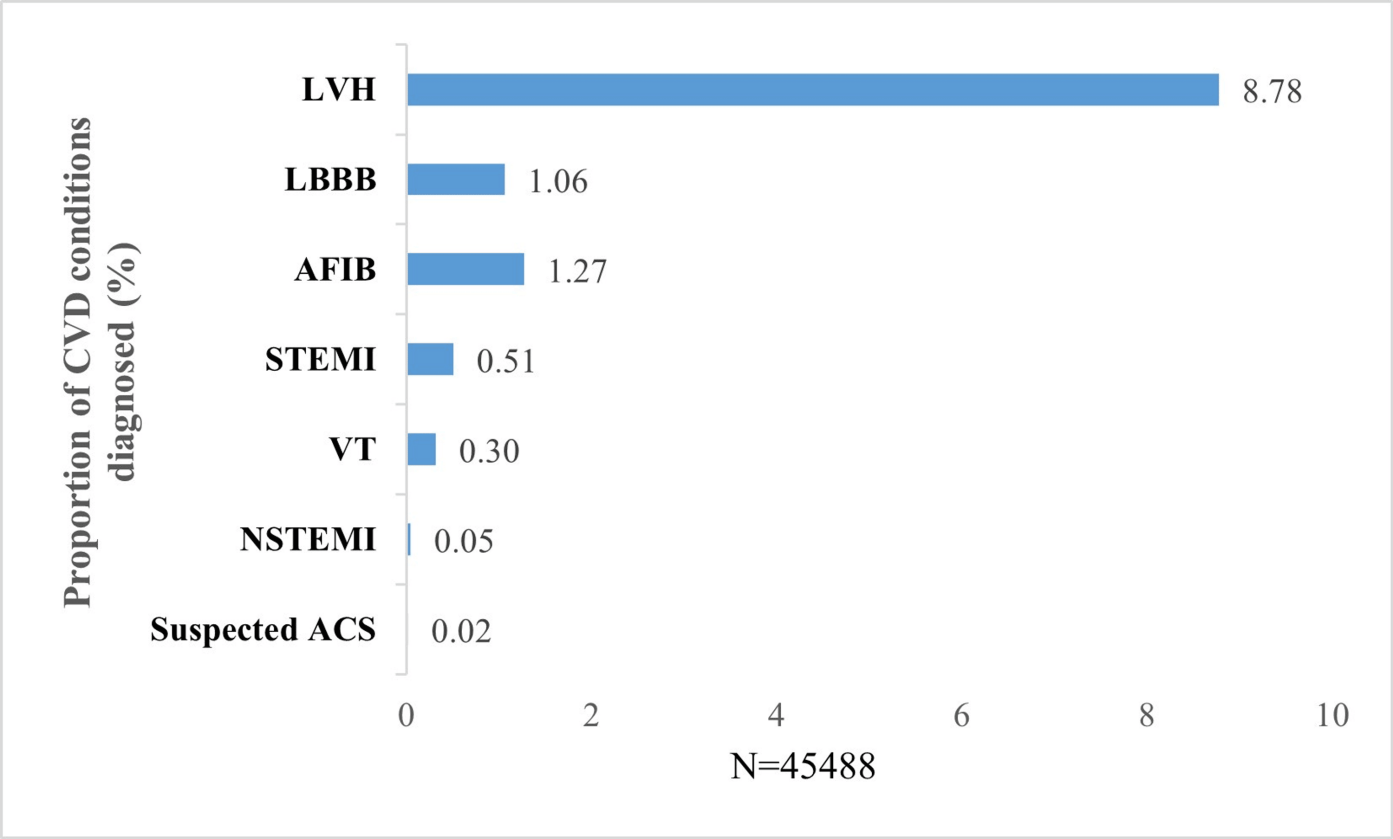
Overall diagnosis of CVD conditions from the collected ECG data ACS, acute coronary syndrome; AFIB, atrial fibrillation; CVD, cardiovascular disorder; ECG, electrocardiogram; LBBB, left bundle branch block; LVH, left ventricular hypertrophy; NSTEMI, non-STEMI; STEMI, ST-elevation myocardial infarction; VT, ventricular tachycardia.

Upon closely analyzing the dataset to identify which ECG category had the maximum number of CVD diagnoses, all seven diagnosed CVD conditions were reported under the Critical ECG category, while four CVD conditions (Suspected ACS, LBBB, STEMI, and LVH) were reported under Abnormal ECG (Table 4). The most frequently reported CVD findings among all ECG categories were LVH (8.78%, 39,93/45,488), LBBB (1.06%, 482/45,488), AFIB (1.27%, 576/45,488), STEMI (0.51%, 231/45,488 of total ECGs), VT (0.30%, 145/45,488), NSTEMI (0.05%, 22/45,488 of total ECGs), and Suspected ACS (0.02%, 7/45,488 of total ECGs) (Figure 1).

**Table 4:** Summary of ECG findings by CVD conditions ACS, acute coronary syndrome; AFIB, atrial fibrillation; CVD, cardiovascular disorder; ECG, electrocardiogram; LBBB, left bundle branch block; LVH, left ventricular hypertrophy; STEMI, ST-elevation myocardial infarction; VT, ventricular tachycardia.

ECG status (N=45488)	Suspected ACS n (%)	LBBB n (%)	AFIB n (%)	VT n (%)	STEMI n (%)	NSTEMI n (%)	LVH n (%)
Abnormal	06 (0.01)	47 (0.10)	00	00	92 (0.20)	00	3540 (7.78)
Borderline	00	00	00	00	00	00	00
Critical	01 (0.00)	435 (0.96)	576 (1.27)	145 (0.32)	139 (0.31)	22 (0.05)	429 (0.94)
Normal	00	00	00	00	00	00	24 (0.05)
Otherwise Normal	00	00	00	00	00	00	00
Pacemaker	00	00	00	00	00	00	00
Total	7 (0.02)	482 (1.06)	576 (1.27)	145 (0.32)	231 (0.51)	22 (0.05)	3993 (8.78)

When analyzing only ACS cases (STEMI+NSTEMI+Suspected ACS, n=260), STEMI accounted for the majority (88.84%, 231/260), followed by NSTEMI (8.46%, 22/260) and Suspected ACS (2.69%, 7/260). The majority of the ACS diagnoses (62.30%, 162/260 (STEMI, 139 + NSTEMI, 22 + Suspected ACS, 1)) were reported from the Critical ECG, while the rest of the 37.69% (98/260 (STEMI, 92 + Suspected ACS, 6)) were reported from the Abnormal ECG (Table 4). Among the Normal ECG category, few cases were diagnosed with LVH (n=24).

The statewide CVD-diagnosed conditions are presented in Table 5. The mean (SD) TAT for all ECGs was 5.12 (9.61) minutes. The mean TAT was minimum for the Critical category (2.91 minutes) and maximum for the Normal ECG category (5.61 minutes) (Table 2).

**Table 5:** Summary of CVD conditions by states (N=45488) ACS, acute coronary syndrome; AFIB, atrial fibrillation; CVD, cardiovascular disorder; LBBB, left bundle branch block; LVH, left ventricular hypertrophy; STEMI, ST-elevation myocardial infarction; VT, ventricular tachycardia.

Hub No.	State	STEMI n (%)	NSTEMI (n (%)	Suspected ACS n (%)	LVH n (%)	LBBB n (%)	AFIB n (%)	VT n (%)
5338	Maharashtra	30 (0.07)	03 (0.01)	00	1141 (2.51)	93 (0.20)	93 (0.20)	12 (0.03)
10497	Maharashtra	33 (0.07)	06 (0.01)	00	673 (1.48)	72 (0.16)	115 (0.25)	13 (0.03)
16147	Haryana	26 (0.06)	01 (0.00)	02 (00)	237 (0.52)	44 (0.10)	38 (0.08)	12 (0.03)
10529	Haryana	41 (0.09)	04 (0.01)	01 (00)	453 (1.00)	54 (0.12)	62 (0.14)	27 (0.06)
16180	Haryana	02 (0.00)	00	00	8 (0.02)	00	02 (0.00)	00
21566	Haryana	13 (0.03)	03 (0.01)	03 (0.01)	222 (0.49)	26 (0.06)	48 (0.11)	09 (0.02)
10197	Madhya Pradesh	40 (0.09)	02 (0.00)	00	602 (1.32)	65 (0.14)	64 (0.14)	32 (0.07)
14805	Rajasthan	11 (0.02)	00	00	135 (0.30)	29 (0.06)	29 (0.06)	07 (0.02)
21578	Uttar Pradesh	32 (0.07)	03 (0.01)	01 (00)	451 (0.99)	90 (0.20)	110 (0.24)	29 (0.06)
16552	Jharkhand	03 (0.01)	00	00	71 (0.16)	09 (0.02)	15 (0.03)	04 (0.01)

## Discussion

The efficacy of the hub-and-spoke model has been well-documented across various healthcare disciplines globally [27-29]. The hub-and-spoke model has enabled one of the Indian states, Tamil Nadu’s STEMI program, to reduce mortality by 3.4% and save INR 193,749 (USD 3,311) per life [30]. The Heart Beat project India utilizes a similar model, incorporating AI assistance alongside human experts for rapid and accurate ECG diagnostics. This inclusion of AI aims to improve diagnostic precision and efficiency, potentially reducing diagnostic delays and shortening time-to-treatment for ACS patients. The current analysis found 42.64% Abnormal and 6.58% Critical ECGs, with the majority of ACS diagnoses (62.30%) being reported from Critical ECGs. When restricted to ACS cases (n=260), STEMI accounted for 88.84% (231/260), followed by NSTEMI at 8.46% (22/260) and suspected ACS at 2.69% (7/260). The average time to generate the AI-assisted, expert-analyzed ECG report was around five minutes. The initial results of the current report indicate that AI-generated ECG reports could facilitate the timely diagnosis of ACS patients, improving their chances of reaching the hospital within the life-saving window. This approach may also help to alleviate the significant burden on the Indian healthcare system by enabling faster intervention and potentially reducing the demand on emergency resources.

The findings from our preliminary data analyses align with the current evidence from global and Indian studies. The recent EPICOR Asia study, which is a multinational, prospective, primary data collection study of real-world management of Asian patients with ACS, reported STEMI in 51.2% and NSTEMI in 19.9% of adult ACS patients [5]. Similarly, studies in Egypt and India (Kerala, North East regions) reported higher STEMI occurrence rates than NSTEMI among ACS cases [31-33]. The CREAT registry found 60% of ACS cases in India were STEMI, a higher proportion than in developed countries [34]. In our Indian population sample (n=260), STEMI constituted 88.84% of ACS cases (231/260), while NSTEMI accounted for 8.46%, highlighting the critical need for rapid STEMI diagnosis to reduce mortality.

LVH is shown to be a major predictor of CVD survival [35]. Its prevalence varies with age, gender, blood pressure, and body mass index (BMI). For instance, the prevalence is around 32% in hypertensive individuals [36] compared to 3% in the general global population [37]. In our Indian chest pain patient population, it was the most common CVD condition at ~9%, but this was lower than India’s previous report of ~15% in the general population [38], warranting further studies for confirmation.

Global AFIB prevalence studies have found a lower prevalence of 1-2% among Indo-Asians compared to Western populations [39,40]. Limited reports from India show a broad range of AFIB prevalence, from 0.1% to 5.1% [41,42]. Our analysis reports a sample prevalence of 1.27%. This variation is likely influenced by age, ethnicity, geographic location, and CV risk; this finding can be further validated through future studies using a dataset generated from the Heart Beat project.

The current findings further align with the available state-level disease burden studies, indicating that the prevalence of IHD and stroke differs considerably among states [43]. While the current analysis was unable to provide precise state-wise differences in CV abnormalities due to uneven sample distribution, with some states contributing more ECG recordings than others, it does offer insights into the overall distribution of CV abnormalities across the Indian states.

The mean TAT for generating ECG diagnosis reports was ~5 minutes in the current analysis. This aligns with the 2022 American College of Cardiology Consensus Report (ACC 2022), which emphasizes the need for ECG performance and interpretation within 10 minutes of a patient's hospital arrival [44]. This AI-assisted TAT reduction has important clinical implications: (1) faster diagnosis-to-treatment cycles for time-sensitive conditions (e.g., STEMI, where door-to-balloon times correlate with mortality); (2) reduced cognitive burden on clinicians during high-volume periods; and (3) potential for improved resource allocation in emergency settings. Moreover, it was also found that Critical ECGs had a faster processing time of 2.91 minutes compared to Normal ECGs at 5.61 minutes, suggesting quicker diagnoses in critical cases. While these TAT improvements are promising, real-world impact depends on system integration factors, including clinician adoption rates, EHR interoperability, and concurrent process optimizations. Future prospective and longitudinal studies are warranted to track patient-level outcomes post-diagnosis, including treatment timelines, mortality, rehospitalization, and healthcare utilization. Although this study did not directly assess mortality or reperfusion timelines, earlier ECG interpretation within established STEMI care pathways has been associated with improved time-to-treatment and survival outcomes.

The findings should be interpreted in light of certain limitations. As a retrospective, observational analysis of programming data, the study is susceptible to confounding and selection bias. The dataset comprises only patients presenting with chest pain at participating centers and therefore does not represent the general population or all individuals undergoing ECG evaluation. Consequently, the reported prevalence of cardiovascular abnormalities reflects a symptomatic chest pain cohort within this network and should not be interpreted as population-level estimates. The uneven geographic distribution of hub-and-spoke centers could limit generalizability, as urban/rural disparities in healthcare access may influence TAT and diagnostic patterns. The study period overlapped with the COVID-19 pandemic (2020-2022), which may have influenced healthcare-seeking behavior, referral pathways, staffing availability, and operational processes. Reduced hospital visits for non-emergent symptoms, altered triage thresholds, and pandemic-related resource reallocation may have affected both the reporting of cardiovascular abnormalities and the measured TAT. The sample size was not determined by a priori power calculations, and reliance on a convenience sample may restrict external validity. While AI-assisted TAT improvements were observed, unmeasured confounders (e.g., concurrent process optimizations) could contribute to the reported reductions.

Although the current report's observational and preliminary nature has limitations, it offers a snapshot of current cardiovascular abnormalities across various Indian regions. Importantly, it highlights the success of AI assistance in reducing ECG diagnosis time in the hub-and-spoke model setup. As additional Heart Beat project data becomes available over time across various regions of the country, longitudinal follow-up studies will be essential, firstly to track patient outcomes post-diagnosis (e.g., treatment adherence, mortality, and quality-of-life metrics), secondly to further elucidate cardiovascular condition prevalence and incidence rates, and finally to assess sustained improvements in DIDO and door-to-balloon times for ACS patients.

Furthermore, future comparative studies with cardiologist overreads will be important for quantifying diagnostic concordance rates and further validating the clinical reliability of the system. These analyses would strengthen the clinical relevance of AI-assisted ECG diagnostics by linking process efficiencies to long-term health outcomes.

## Conclusions

This analysis of 45,488 patients across six Indian states demonstrates the clinical utility of an AI-assisted hub-and-spoke model in improving ACS diagnosis. The observed predominance of STEMI emphasizes the critical need for rapid detection, a need addressed by the AI platform, which achieved a mean TAT of 2.91 minutes for critical ECGs, significantly below the ACC-recommended 10-minute benchmark for timely ECG evaluation. By facilitating faster identification and triage of high-acuity ECG patterns, this system directly supports time-sensitive interventions (e.g., door-to-balloon workflows) while optimizing resource allocation at spoke centers. Future studies should correlate these TAT improvements with vital outcomes (e.g., mortality reduction) and explore AI’s role in mitigating geographic disparities in cardiac care.
